# Concentration, Size Distribution, and Infectivity of Airborne Particles Carrying Swine Viruses

**DOI:** 10.1371/journal.pone.0135675

**Published:** 2015-08-19

**Authors:** Carmen Alonso, Peter C. Raynor, Peter R. Davies, Montserrat Torremorell

**Affiliations:** 1 Department of Veterinary Population Medicine, College of Veterinary Medicine, University of Minnesota-Twin Cities, Saint Paul, Minnesota, United States of America; 2 Division of Environmental Health Sciences, School of Public Health, University of Minnesota-Twin Cities, Saint Paul, Minnesota, United States of America; University of Georgia, UNITED STATES

## Abstract

When pathogens become airborne, they travel associated with particles of different size and composition. Particle size determines the distance across which pathogens can be transported, as well as the site of deposition and the survivability of the pathogen. Despite the importance of this information, the size distribution of particles bearing viruses emitted by infectious animals remains unknown. In this study we characterized the concentration and size distribution of inhalable particles that transport influenza A virus (IAV), porcine reproductive and respiratory syndrome virus (PRRSV), and porcine epidemic diarrhea virus (PEDV) generated by acutely infected pigs and assessed virus viability for each particle size range. Aerosols from experimentally infected pigs were sampled for 24 days using an Andersen cascade impactor able to separate particles by size (ranging from 0.4 to 10 micrometer (μm) in diameter). Air samples collected for the first 9, 20 and the last 3 days of the study were analyzed for IAV, PRRSV and PEDV, respectively, using quantitative reverse transcription polymerase chain reaction (RT-PCR) and quantified as geometric mean copies/m^3^ within each size range. IAV was detected in all particle size ranges in quantities ranging from 5.5x10^2^ (in particles ranging from 1.1 to 2.1μm) to 4.3x10^5^ RNA copies/m^3^ in the largest particles (9.0–10.0μm). PRRSV was detected in all size ranges except particles between 0.7 and 2.1μm in quantities ranging from 6x10^2^ (0.4–0.7μm) to 5.1x10^4^ RNA copies/m^3^ (9.0–10.0μm). PEDV, an enteric virus, was detected in all particle sizes and in higher quantities than IAV and PRRSV (p < 0.0001) ranging from 1.3x10^6^ (0.4–0.7μm) to 3.5x10^8^ RNA copies/m^3^ (9.0–10.0μm). Infectious status was demonstrated for the 3 viruses, and in the case of IAV and PRRSV, viruses were isolated from particles larger than 2.1μm. In summary, our results indicated that airborne PEDV, IAV and PRRSV can be found in a wide range of particle sizes. However, virus viability is particle size dependent.

## Introduction

Among all infectious agents, those transmitted through aerosols are the most difficult to control [1]. The speed of dispersion of airborne infectious agents makes them hard to contain and protect against, and the wide reach of susceptible hosts makes the control of airborne pathogens a priority for public and animal health officials.

Infectious agents travel associated with particles of various natures including fecal material, dust, debris, water, respiratory fluids and, specifically in buildings housing animals, with bedding and hair particles. The composition and size distribution of these particles will determine the location of deposition in the susceptible host, and influence the time the infectious agents can remain suspended in the air, the distance across which they can be transported, and the survivability and infectivity of the pathogens [2–4]. Thus, particle size is central to the epidemiology of airborne pathogens.

Particles of small size can remain suspended in the air for long periods, potentially exposing a large number of susceptible individuals, including those close to the source and those at greater distances [5]. A spherical particle of 4 μm in diameter takes 33 min to settle 1 m in still air, compared to a 1μm particle that will take 8 h [6]. Relative humidity, temperature and wind currents are the most important environmental factors that will determine the settling time of airborne particles that contain volatile components.

There is limited information in regards to the particle sizes with which infectious agents are associated. In humans, measures of particle size for influenza A virus (IAV) have been evaluated in controlled laboratory and health care settings, airplanes, daycares and households [6–10]. Distribution of IAV particle size varied between studies and settings, with IAV found in particles 1–4 μm in diameter (49% of particles) and particles > 4 μm (46% of particles) in health care facilities [7], and particles < 2.5 μm (64% of viral copies) detected in public places (i.e. health care, airplanes and day cares) [8]. Furthermore, dispersion models that have incorporated particle size information have indicated the plausibility of airborne transmission within these settings [3].

For pigs and cattle, the size distribution of particles associated with foot and mouth disease virus (FMDV) depended on whether aerosols were generated artificially or by experimentally infected animals [11,12]. In the case of pigs affected by Aujeszky’s disease virus (ADV), particle size distribution varied based on days of infection [13]. Specifically for IAV, no information was found for animals raised in agricultural environments, and the information is limited to animal models using ferrets or guinea pigs that assess the risk of IAV airborne transmission to people [14–16]. Overall, there is a lack of information on particle size distribution for pathogens affecting humans and animals, including zoonotic viruses. Furthermore, studies have been limited to viruses affecting the respiratory tract even though there is evidence that enteric viruses [i.e porcine epidemic diarrhea virus (PEDV), human-noroviruses, adenoviruses and enteroviruses] can also be transmitted through the air [17–20].

In this study we characterized the particle concentrations, size distributions and infectivity of three viruses that affect swine. These viruses were selected because of their differences in pathogenesis and modes of transmission. IAV, important because of its zoonotic potential, is shed only in respiratory secretions of pigs, and it is well established that it can be transmitted through aerosols. Porcine reproductive respiratory syndrome virus (PRRSV) is a systemic virus that can be shed in many body secretions [21], is exhaled in air, and has been detected in air as far as 9.1 km from swine herds [22]. Lastly, PEDV, an emerging virus in North America [23], is an enteric virus that primarily replicates in large quantities in the small intestine, is largely concentrated in feces of diarrheic pigs and can replicate in alveolar macrophages [24]. The information from this study may contribute to the understanding of airborne transmission of viruses of different pathogenesis and routes of transmission, which is necessary to fully prevent the spread of infectious diseases.

## Materials and Methods

### Ethics statement

Protocols and procedures followed throughout the study were approved by the University of Minnesota Institutional Animal Care and Use Committee (IACUC1110A05802), and the Institutional Biosafety Committee (IBC1208H18341).

### Housing and experimental animals

The study was performed at the University of Minnesota BSL-2 research animal units, St. Paul, MN. The room has a total air space of 35.1 m^3^ and the air entering the isolation room was filtered. Relative humidity and temperature were monitored continually using a data logger (ThermaData Logger-Model HTD, ThermoWorks, Lindon, UT, US). A group of twelve, 5-week-old pigs were purchased from a herd that tested negative for IAV, PRRSV, PEDV and *Mycoplasma hyopneumoniae* on routine serologic and antigenic testing. The negative status of the experimental pigs was confirmed prior to inoculation. Serum samples were tested for IAV and PRRSV via enzyme linked immunosorbent assays (ELISA) (IDEXX AI Ab Test and HerdChek X3 ELISA, IDEXX, ME, US) [25,26]. Nasal swabs and rectal swabs were tested for IAV and PEDV respectively using real-time reverse transcriptase PCR (RT-PCR) [17,27]. Microchips were subcutaneously implanted to record body temperature following manufacturer specifications (LifeChip, Destron Fearing, MN, US). Pigs were housed in solid floors without bedding and fed once a day on the floor. Water was provided *ad libitum*.

### Virus inoculation

Ten pigs out of 12 were inoculated with PRRSV and IAV 48 h after arrival at the isolation units. Two pigs were removed from the room prior to inoculation and commingled back with the rest of the pigs 6 hours after to serve as contact infected controls. Pigs were sedated using an intramuscular injection of Telazol (Fort Dodge Animal Health, Fort Dodge, IA, USA) at 6mg/Kg, and experimentally inoculated intra-nasally and intra-tracheally with 1 ml/each of 4.4 x 10^6^ tissue culture infective dose (TCID_50_/ml) of IAV/Swine/Iowa/00239/2004 H1N1. Following that, pigs were inoculated intra-muscularly and intra-nasally with 1 ml/each of 1.13 x 10^5^ TCID_50_/ml of PRRSV strain MN-1-8-4 [28,29].

At day 21 of the study, all pigs were intra-gastrically inoculated with a suspension of 20 ml of PEDV-material obtained from mucosal scrapings from PEDV infected pigs following published procedures [17]. The inoculation material was confirmed positive by PEDV RT-PCR and diluted to a cycle threshold (Ct) value of 15 to 16. The inoculation material was prepared and kept refrigerated for 24h at 4°C prior to inoculation. The total duration of the study was 24 days.

### Clinical scores and body temperature

Body temperature and clinical signs were recorded twice daily starting 72 hours pre-inoculation. Fever was defined as rectal temperature higher than 40°C for 2 or more consecutive days. Clinical scores included lethargy and signs of respiratory disease (i.e. coughing and sneezing episodes). Lethargy score was defined *a priori* on a 1 to 5 scale and measured based on the response to clapping or flight reaction, and curiosity of the pigs towards the investigator and sampling rope (**S1 Table**). This procedure was repeated 3 times each day and final scores were averaged. The respiratory disease score was defined as the number of pigs with coughing and/or sneezing episodes (i.e. one or several episodes in a sequence by an individual pig) registered in 3 minutes. The respiratory score was calculated as a percent by dividing the number of animals with coughing and/or sneezing episodes by the total number of animals observed and multiplying the result by 100 [30].

### Sampling procedures

Nasal swabs were collected for IAV testing from all pigs on -2, 3, 5, 7, 9 and 13 days post infection (DPI) using rayon-tipped swab applicators with Stuart’s medium (BBL CultureSwabs liquid Stuart single plastic applicator/Becton, Dickinson and Com., Sparks, Maryland, USA). After sample collection, swabs were transported to the laboratory, suspended in 2 ml of minimal essential medium (MEM Mediatech Inc., Manassas, VA, USA) supplemented with 4% of bovine serum albumin (BSA) and stored at -80°C.

Serum samples for PRRSV viremia were collected using venipuncture of the jugular vein at days -2, 4, 12 and 20 DPI. After collection, serum was separated, divided into 2 aliquots and stored at -80°C.

Oral fluids for PRRSV and IAV were collected daily at the same time when air samples were collected by hanging 0.4 m of cotton rope from a convenient metallic post following published procedures [31,32]. Oral fluid samples were then refrigerated, transported to the laboratory and stored at -80°C. For PEDV, at the termination of the study, intestines were removed from all animals and sections from 6 different areas of the jejunum and ileum were collected for histopathology.

Total particle concentrations and size distributions were measured using an optical particle counter (OPC) (AeroTrak 9306 Handheld Particle Sizer, TSI, Inc., St. Paul, MN) able to separate particles from 0.3 to >10 μm into 6 size intervals. To measure airborne virus concentrations, samples were collected twice daily (9:00 a.m. and 3:00 p.m.) for 17 days and once (9:00 a.m.) for 6 days using both a liquid cyclonic collector (Midwest Micro-tek, Brookings, SD, USA) [29] and an Andersen cascade impactor (ACI; Thermo Electron Corporation, Waltham, MA, USA) [33], which is able to separate particles according to size. The capability of both air collectors for quantifying the presence of virus was compared. Briefly, the 2 air samplers were positioned approximately 1.3 m height from the floor and the OPC was positioned 1.7 m from the floor. The distance between the air samplers was 0.9 m. The pigs did not have direct contact or access to the devices.

Sample collection using the cyclonic air collector (which was demonstrated to process 200 l of air per minute) was carried out for 30 min. Ten milliliters of MEM supplemented with 4% of bovine albumin serum were used as collection media. After collection, an average of 4 ml of sample was recovered, divided into 2 aliquots, and stored at -80°C. The collector was then disinfected with 70% ethanol (or 10% chlorine during PEDV air sampling), rinsed with distilled water and dried with paper towels. After disinfection, the collection vessel and the turbine were swabbed, and samples stored at -80°C until analysis. The ACI sampled air at 28.3 l/min for 1 hour, and separated particles into 8 size intervals: 0.4–0.7, 0.7–1.1, 1.1–2.1, 2.1–3.3, 3.3–4.7, 4.7–5.8, 5.8–9.0 and >9.0 μm. Samples from this device were eluted from every plate stage using a cell scraper and 1 ml of MEM [33]. All samples were transferred into 1.5 ml sterile plastic tubes, placed on ice and stored at -80°C until testing. The ACI was then disassembled and plates and stages were scrubbed and disinfected with alkyl dimethyl benzyl ammonium chloride soap (Lysol, Reckitt Benckiser) and finally rinsed and dried with paper towels. After disinfection, a minimum of 4 collection plates and individual ACI stages were swabbed, and samples stored at -80°C. For each air sampling event, there were 8 stages assayed for the ACI and 1 sample for the cyclonic air collector.

### Laboratory procedures

Oral fluid and air samples were tested using quantitative PRRSV, IAV and PEDV RT-PCRs as previously described [17,27,34]. The samples from day 1 to day 9 DPI were tested for IAV quantitative RT-PCR; for PRRSV from day 1 to day 20 DPI; and for PEDV from DPI 22 to 24 of the study. Nasal swabs, serum samples and intestinal/fecal swab samples were tested for IAV, PRRSV, and PEDV semiquantitative RT-PCRs respectively. For all three viruses, RT-PCR Ct values <35 were considered positive, 35–40 suspect, and > 40 negative.

To assess the infectivity of the air samples, virus isolation was attempted from all RT-PCR positive and suspect air samples in Madin-Darby canine kidney (MDCK) cells for IAV, MARC145 for PRRSV and VERO cells for PEDV using published procedures [35–37]. Furthermore, because virus isolation is typically unsuccessful for North American variants of PEDV, a bioassay was performed by inoculating three susceptible piglets with air samples that tested PEDV RT-PCR positive. Briefly, four 10-day-old pigs from a PEDV-negative farm were purchased and each pig allocated to a separate isolation room. Each pig was intra-gastrically inoculated as described earlier in methods with PBS only (negative control) or 2 ml of pooled air samples containing the collection media of the cyclonic air collector diluted 1:10 with PBS to obtain a total of 20 ml of inoculation material per pig. All pigs were euthanized 4 days post-exposure by injection of 2 ml of pentobarbital (Fatal-Plus, 100 mg/kg IV) into the external jugular vein. Histomorphology analysis was then performed.

### Statistical analysis

Data from the quantitative RT-PCR results, OPC by sampling day, type of sample and pathogen were consolidated in a spreadsheet (Microsoft EXCEL; Microsoft Corporation, Redmond, Washington, USA) and organized for analysis. Means, standard deviations, and minimum and maximum values for quantitative variables, and frequency counts and percentages for qualitative variables were calculated for descriptive analysis. Considering day as a repeated measure and particle size as an independent variable, the quantity of virus (RNA copies) for each particle size per m^3^ of air and mean concentration difference across all three viruses per size interval were assessed for significance using a repeated measures regression model in SAS 9.1 (SAS Institute, Cary, North Carolina, USA). Negative days (considered those of which results from both collectors were negative) were not included in the analysis. An ACI sample was included in the analysis when one or more samples from the eight stages of the sampler had a Ct value within the positive or suspect ranges. Tukey’s method was also used in conjunction with ANOVA for pairwise comparison of virus concentrations among all particle size intervals.

## Results

### Environmental conditions, clinical signs and pig infection results

All inoculated and contact pigs became infected. For IAV all inoculated pigs, and contact controls became positive by 3 and 7 DPI (Ct values 24.6 ± 2.21 and 21.1 ± 2.79) respectively. For PRRSV all inoculated pigs and 1 of the 2 contact pigs were viremic at 7 DPI, and all pigs remained positive until 20 DPI.

**Fig 1:**
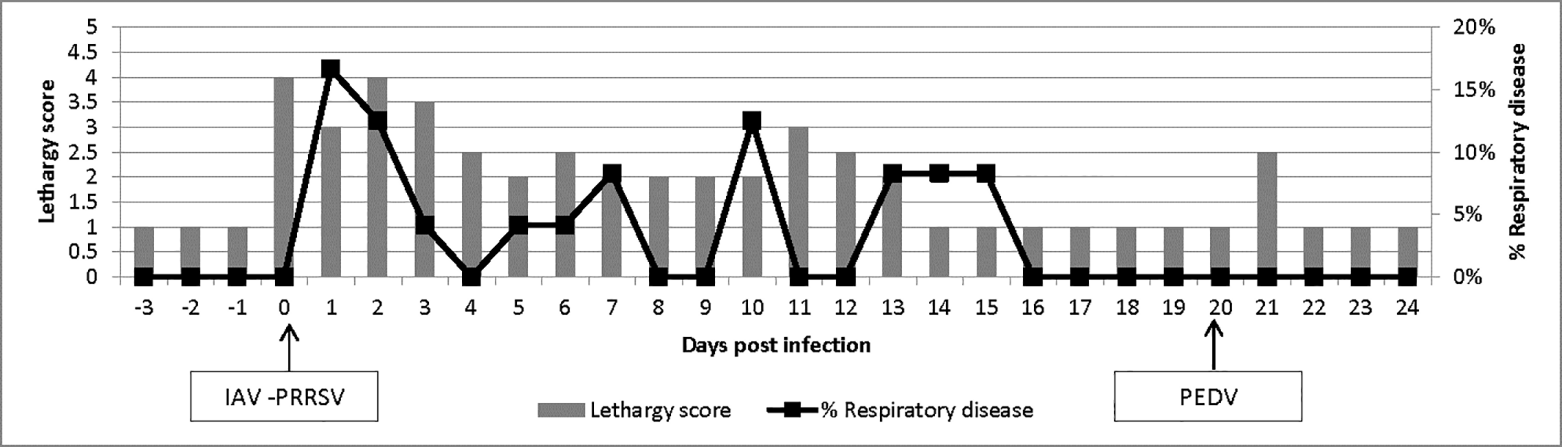
Clinical scores. Clinical scores of lethargy (1 to 5 scale) and percentage of respiratory disease (number of pigs with coughing and/or sneezing episodes) throughout the study.

Animals had an average body temperature of 39.3 ±0.6°C (mean ± standard deviation) and, an average lethargy and respiratory score of 1.98 ± 0.98 and 3.5% ± 0.05% respectively across the entire study. For the first 4 days after infection with IAV and PRRSV, animals had the maximum lethargy and respiratory scores of 4 and 16.7%, respectively (**Fig 1**). Animals recovered from any respiratory clinical signs by 16 DPI. Lethargy scores ranged between 0 and 1 for the remainder of the study, except for the day after PEDV infection that increased to 2.5. All pigs had diarrhea 48h after PEDV inoculation.

The mean indoor temperature (mean ± standard deviation) was 23.2°C±0.7°C with a mean relative humidity of 32.6% ±7.4% and a mean air pressure of -27.4 Pa ± 0.15Pa relative to the hallway pressure.

### Viral load and viral viability in air samples

**Fig 2:**
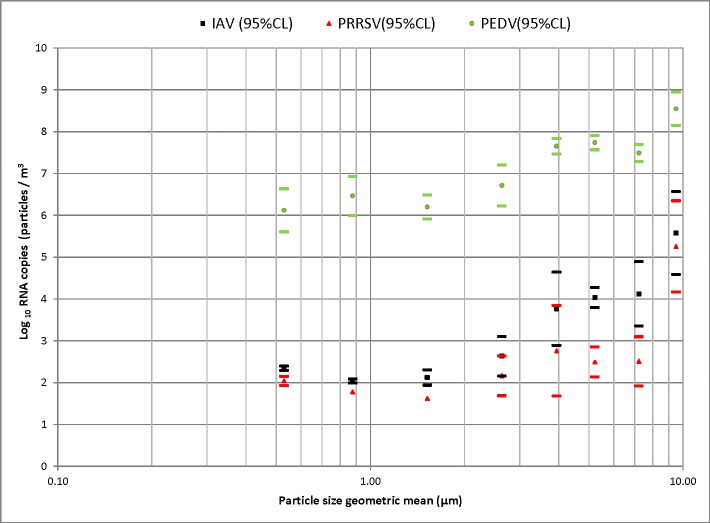
Particle size distribution of airborne viruses. Particle size distribution (Least Squares Means of log10 RNA copies/m3 of air and 95% confident interval) for influenza, porcine reproductive and respiratory syndrome and porcine epidemic diarrhea viruses detected by the Andersen cascade impactor from aerosols generated by infected pigs.

IAV RNA was detected by RT-PCR in air samples collected at 36h post-infection and until 9 DPI. A total of 58.3% (70/120) ACI stages and 93.3% (14/15) samples from the cyclonic collector tested IAV RT-PCR positive. All negative control samples tested negative. IAV RNA was detected in particles of all size ranges tested (**Fig 2**). However, there was higher viral load of IAV in larger particles (> 9 μm) compared to smaller ones (≤9 μm) (*p* < 0.001) (**Table 1**).

**Table 1:** Distribution by particle size of the quantity [geometric mean of RNA copies/m^3^ (geometric standard deviation)] of influenza (IAV), porcine reproductive and respiratory syndrome (PRRSV) and porcine epidemic diarrhea (PEDV) viruses in air samples.

Particle size range (μm)	IAV	PRRSV	PEDV
0.4–0.7	8x10^2^ (3.2) ^a^	6x10^2^ (4.1) ^a^	1.3x10^6^ (3.4) ^a^
0.7–1.1	6.1x10^2^ (1.7) ^a^	^†^≤5.1x10^2^ (1) ^a^	3x10^6^ (4.7) ^a^
1.1–2.1	5.5x10^2^ (2.5) ^a^	^†^≤3.6x10^2^ (1) ^a^	1.6x10^6^ (2.4) ^a^
2.1–3.3	1.4x10^3^ (4) ^a^ ^,^ ^b^	7.8x10^2^ (4.82) ^a^	5.2x10^6^ (3.3) ^a^ ^,^ ^b^
3.3–4.7	7.8x10^3^ (5.1) ^b^ ^,^ ^c^	1.8x10^3^ (1.26x10^1^) ^a^	4.5x10^7^ (1.8) ^c^
4.7–5.8	2.3x10^4^ (7.4) ^c^	1.7x10^3^ (4.8) ^a^	5.5x10^7^ (2.2) ^c^ ^,^ ^d^
5.8–9.0	1.5x10^4^ (6) ^c^	1.1x10^3^ (7) ^a^	3.1x10^7^ (2) ^b^ ^,^ ^c^
> 9.0	4.3x10^5^ (1.25x10^1^) ^d^	5.1x10^4^ (2.8x10^1^) ^b^	3.5x10^8^ (2.9) ^d^

^a, b, c, d^ Different superscripts between rows of the same column indicate statistically significant differences (Tukey’s test, *p* < 0.05)

^†^LOD^:^ Limit of q-PCR detection

Using the cyclonic air collector, IAV RNA was detected in the air for 8 DPI and the total geometric mean (and geometric standard deviation) viral concentration was similar [9.1x10^4^ (1.3x10^1^)] to the ACI [4.77x10^4^(4.07)] (*p* = 0.403). IAV was isolated in cell culture from 28.6% (20/70) of ACI samples and 35.7% (5/14) of air cyclonic samples, and from particles of all sizes > 2.1 microns (**Table 2**).

**Table 2:** RT-PCR and virus isolation results by particle size and air sampler type. Number of positive RT-PCR and virus isolation (VI) air samples for influenza (IAV), porcine reproductive and respiratory syndrome (PRRSV) and bioassay for porcine epidemic diarrhea (PEDV) viruses collected from acutely infected animals. Results are presented by air sampler, and in the case of the Andersen cascade impactor, by particle size rage.

Virus		Andersen cascade impactor (ACI) (particle size ranges in μm)									
		0.4–0.7	1.1–0.7	1.1–2.1	2.1–3.3	3.3–4.7	4.7–5.8	5.8–9.0	9.0–10.0	Total ACI	Total cyclonic collector
IAV	^1^PCR	*6/15 (40.0%)	3/15 (20.0%)	5/15 (33.3%)	6/15 (40.0%)	12/15 (80.0%)	12/15 (80.0%)	13/15 (86.7%)	13/15 (86.7%)	70/120 (58.3%)	14/15 (93.3%)
	^2^VI	0/6	0/3	0/5	1/6 (16.7%)	1/12 (8.33%)	6/12 (50.0%)	4/13 (30.8%)	8/13 (61.5%)	20/70 (28.6%)	5/14 (35.7%)
PRRSV	PCR	1/34 (2.9%)	0/34	0/34	1/34 (2.9%)	2/34 (5.9%)	1/34 (2.9%)	2/34 (5.9%)	7/34 (20.6%)	14/272 (5.1%)	8/34 (23.5%)
	VI	0/1	0	0	1/1 (100%)	2/2 (100%)	1/1 (100%)	0/2 (0%)	7/7 (100%)	11/13 (84.6%)	6/8 (75%)
PEDV	PCR	6/6 (100%)	6/6 (100%)	6/6 (100%)	6/6 (100%)	6/6 (100%)	6/6 (100%)	6/6 (100%)	6/6 (100%)	48/48 (100%)	6/6 (100%)
	Bioassay	^†^NA	NA	NA	NA	NA	NA	NA	NA	NA	^§^3/3 (100%)

^1^PCR: Reverse transcription polymerase chain reaction results (includes samples with cycle threshold (Ct) value less than 40)

^2^VI: Virus isolation results

*Number of positive samples out of total number of samples tested (%)

^†^NA: Not applicable

^§^Bioassay results were obtained from the inoculation of susceptible pigs with RT-PCR positive air samples using the air cyclonic collector

PRRSV RNA was detected for the first time in air samples collected at 7DPI and until 17 DPI, on a total of 8 days. A total of 5.1% (14/272) samples from the ACI stages and 23.5% (8/34) from the cyclonic collector tested RT-PCR positive. Negative results from ACI stages collected during 12 days of the study were not considered for the viral load analysis. All negative control samples tested negative.

PRRSV RNA was detected in particle sizes ranging from 0.3 to 0.7 μm and in particles ranging from 2.1 to 10 μm (**Fig 2**). However, there was higher viral load of PRRSV in larger particles (0.9–10 μm) compared to smaller ones (<9 μm) (*p* = 0.015) (**Table 1**).

There was no difference in geometric mean concentration of PRRSV RNA in air samples collected with the cyclonic collector [5x10^3^ (3.8x10^1^)] versus the ACI [1.5x10^4^ (1.1x10^1^)] (*p* = 0.353). PRRSV was isolated in cell culture from 78.6% (11/14) ACI samples and 75% (6/8) air cyclonic samples, but only in samples from particles > 2.1 microns (**Table 2**).

PEDV RNA was detected in all air samples collected from 24 hours post infection to the termination of the study (**Fig 2**). All negative control samples tested negative.

PEDV RNA was detected in particles of all size ranges tested but there was higher concentration in particles >3.3 to 10 μm compared to those in ranges 0.4 to 3.3 μm. No difference in geometric mean viral concentration was observed between samples from the cyclonic air collector [9.1x10^7^(2.7)] versus the ACI [4.5x10^7^(1.8)] (*p* = 0.1601). PEDV could not be isolated by standard cell culture techniques. However, all bioassay pigs infected with air samples experienced moderate to severe diarrhea, shed high quantities of PEDV in feces ranging from 3.96 x 10^10^ to 7.57 x 10^10^ (RNA copies/ml, Ct 15–16), and had histopathological lesions of moderate to marked atrophic enteritis compatible with PEDV infection. The negative control pig showed no clinical signs, tested negative by PCR and had normal intestinal histomorphology.

Overall there were higher concentrations of airborne PEDV compared to PRRSV and IAV (*p* < 0.0001) associated with each particle size, and higher concentrations of IAV compared to PRRSV in large particle sizes ranging from 4.7 to 9 μm (*p* < 0.05) (**Fig 2**).

### Total particles by size

**Fig 3:**
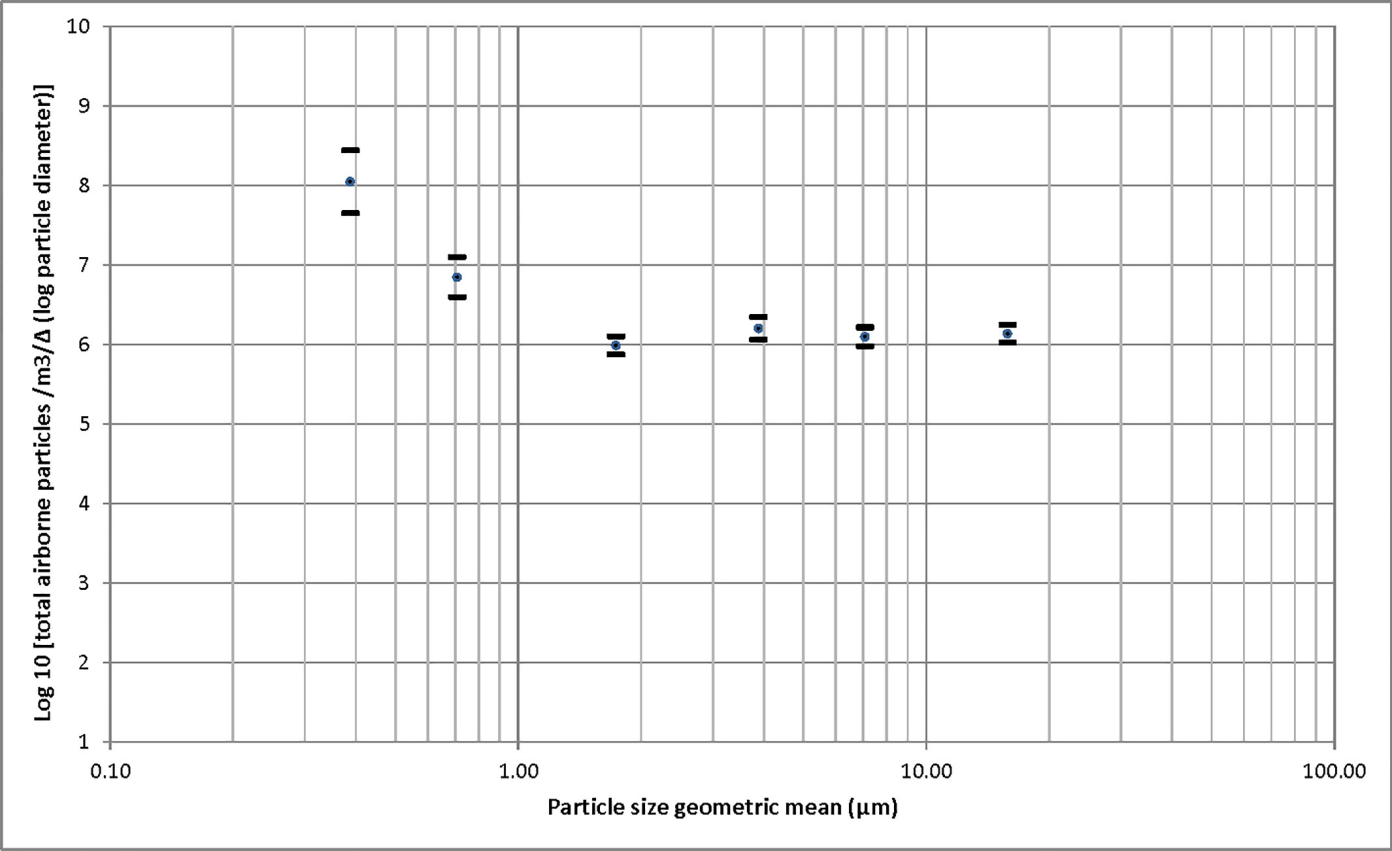
Total airborne particles distribution. Distribution of total airborne particles (geometric mean of number of particles/m^3^ and 95% confident interval) measured using an optical particle counter.

An optical particle counter was used to measure the size distribution of total particles in the air for the 24 days of the study (**Fig 3**). There were more total particles within the first submicrometer size (0.3–0.5 μm) compared to particles larger than 0.5 μm (*p* < 0.05). There were no statistically significant differences in the distribution of particles sizes among days throughout the study (results not shown).

## Discussion

We investigated the particle concentration, size distribution, and infectivity of three animal viruses with different pathogenesis and transmission routes. IAV, PRRSV and PEDV emitted by infected pigs were found associated with a wide range of particle sizes that can deposit throughout the respiratory tract and later be swallowed [18]. However, virus viability was particle size dependent with IAV and PRRSV isolated only from particles larger than 2.1 μm. PEDV, primarily an enteric virus, was found in larger concentrations than PRRSV and IAV while suspended in the air. Our results support the relevance of the aerosol route in the transmission of IAV, PRRS and PED viruses.

Viruses transmit differently based on the routes of excretion and ports of entry in susceptible hosts. The viruses investigated in this study have been shown to be infectious while found airborne [17,29,38]. Our study indicated that virus-associated particles disperse simultaneously across a wide range of particle sizes. This is important because it shows that viruses in airborne particles emitted or generated by animals can be transmitted simultaneously across both short and long distances. Although various conditions, including environmental temperature and relative humidity can affect the size distribution of aerosols, the findings from this study are relevant given that the viruses were emitted by pigs housed under environmental conditions similar to those found in environments where pigs and people interact.

Higher quantities of IAV, PRRSV and PEDV were found associated with larger particles, and in the case of IAV and PRRSV virus viability was also associated with larger particle size. Viability of PEDV based on particle size could not be assessed because of the lack of protocols to grow PEDV in cell culture at the time of the study, although viability in air samples was confirmed by bioassay. The association of increasing virus infectivity and concentration, with increasing particle size, has been reported before, and it has been shown to follow a power law relationship greater than 3 [4]. The bigger the particle, the higher the probability that it will carry virus and be infectious. In this study, viability was only shown for PRRSV and IAV in particles bigger than 2.1 μm in diameter, indicating that measures should be considered to mitigate transmission across both short and long distances.

Determining the particle size distribution for both respiratory and enteric viruses has important implications for the control of animal and human diseases and the use of droplet and airborne infection control measures. In general, it is considered that only respiratory viruses are airborne despite some limited evidence that enteric viruses, such as adenovirus, norovirus or enteroviruses, can be airborne [17–20]. Infectious particles sized less than 10 μm tend to have more serious health implications as they are able to penetrate into the lower respiratory tract to establish infection, and in the case of enteric viruses they may deposit in the upper respiratory tract (i.e. tonsils) or the enteric tract by inhalation.

Interestingly, the quantity of PEDV in airborne particles was higher than that for PRRSV and IAV indicating the complexity of airborne transmission and that other factors such as quantity shed (i.e. course of infection), volume of secretions or excretions (i.e. mucus, feces, vomits), age of the animals, type and severity of clinical signs, and type of housing and air flows need to be taken into consideration when the risk of airborne transmission is being evaluated. In this case, we speculate that both, the quantity of PEDV shed per gram of feces in acutely infected pigs, as well as the volume of liquid found in the diarrheic material may explain the higher quantities of RNA copies of PEDV compared to IAV or PRRSV. Furthermore, comprehensive personal protective equipment including respiratory protection should be considered for potential exposures to both respiratory and enteric viruses, in particular in settings where animals and people interact. In addition, other biosecurity measurements such as air filtration could be considered to protect nearby at risk populations as previously demonstrated [39].

The information generated in this study is especially important to design effective airborne disease control programs for both enteric and respiratory viruses, including mitigation of occupational exposure of zoonotic pathogens. Changes in recommendations to protect from airborne viruses should be considered based on exposure to particles of different sizes.

## Supporting Information

S1 Table Lethargy score table. Scores based on the combination of two parameters: the response to clapping or flight reaction, and the curiosity of the pigs towards the investigator and sampling rope
(TIFF)
