# Supplementary figures and images for: Concentration, Size Distribution, and Infectivity of Airborne Particles Carrying Swine Viruses
Source: PLoS One. 2015 Aug 19;10(8):e0135675. doi: 10.1371/journal.pone.0135675 (PMC4545937; doi:10.1371/journal.pone.0135675)

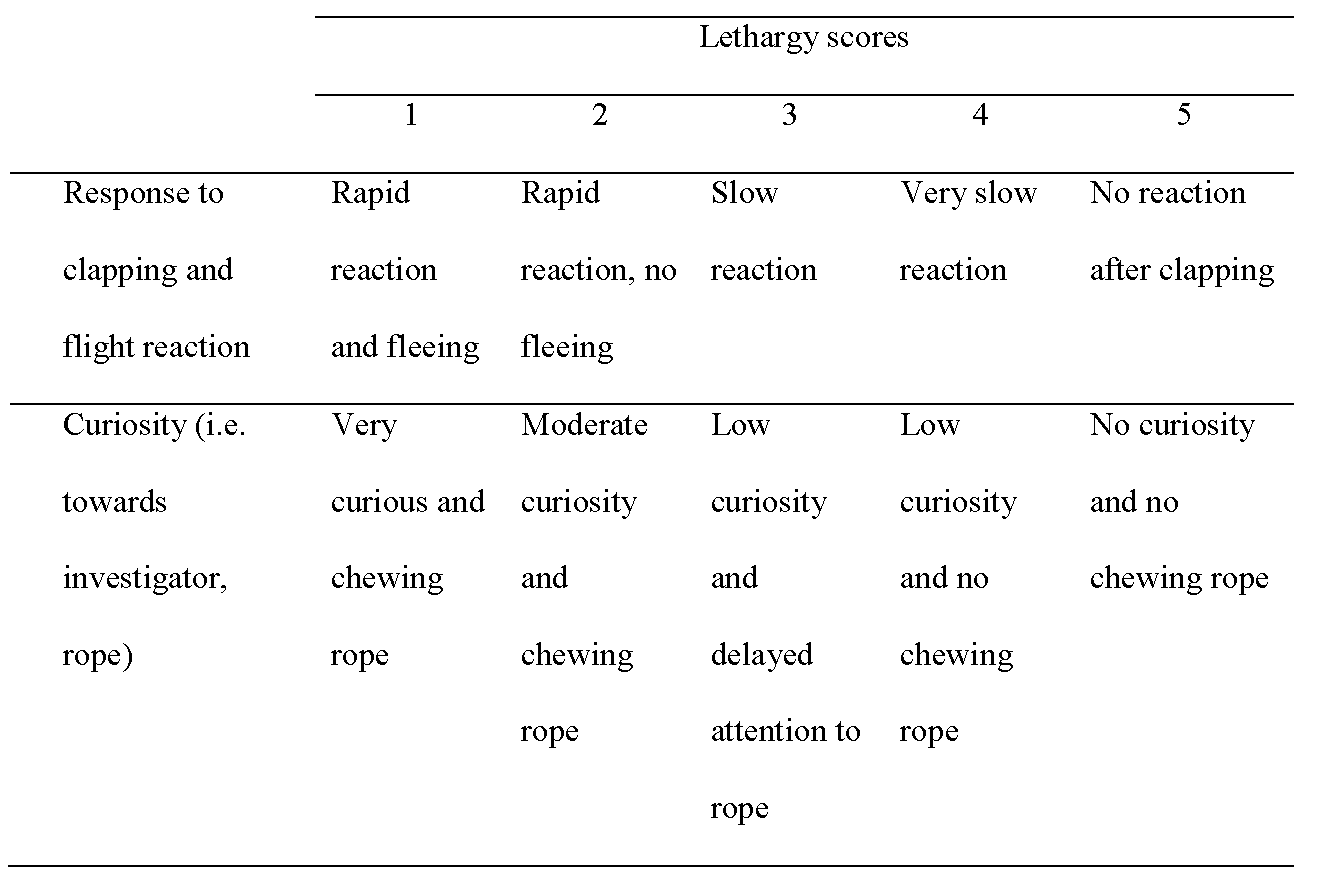

Supplement: S1 Table — Scores based on the combination of two parameters: the response to clapping or flight reaction, and the curiosity of the pigs towards the investigator and sampling rope (TIFF) [file pone.0135675.s001.tiff]
